# Does Personal Goal Pursuit Alleviate Family Caregiver Stress?

**DOI:** 10.1093/geroni/igab046.1074

**Published:** 2021-12-17

**Authors:** Shelbie Turner, Karen Hooker

**Affiliations:** 1 Oregon State University, Oregon State University, Oregon, United States; 2 Oregon State University, Corvallis, Oregon, United States

## Abstract

Family caregivers may experience reduced stress by maintaining their sense of self throughout their time in a caregiving role. Working towards personal goals is helpful for maintaining a sense of self, but pursuing one’s own goals amidst caregiving responsibilities may be challenging. In this study, we analyze the processes by which caregivers pursue their own personal goals – and how those processes impact daily stress – in an effort to develop a deeper understanding of goal-pursuit as a potential caregiver stress-reducing strategy. We utilized daily data from spousal (N=256 days) and adult-child (N=400 days) caregivers who participated in the PULSE (Personal Understandings of Life and Social Experiences) Project, a 100-day microlongitudinal study on goal pursuit amongst people 50 and older (Hooker et al., 2013). In daily surveys, caregivers reported progress made towards a personally-identified health and social goal, along with a 4-item measure of daily stress. We ran multi-level models to assess how daily goal progress was associated with same-day stress. Spousal caregivers’ daily stress was lower on days when their health goal (Estimate = -1.07, SE = 0.20, p<.0001) and social goal (Estimate = -0.97, SE = 0.15, p<.0001) progress was higher. Similarly, adult-child caregivers’ daily stress was lower on days when their health goal (Estimate = -0.67, SE = 0.19, p<.001) and social goal (Estimate = -0.52, SE 0.24, p=0.03) progress was higher. Results support the hypothesis that maintaining personally-meaningful goals can alleviate caregiver stress, and is a promising tool for caregiver health promotion.

